# Newborn Screening for Sickle Cell Disease: Technical and Legal Aspects of a German Pilot Study with 38,220 Participants

**DOI:** 10.1155/2014/695828

**Published:** 2014-07-23

**Authors:** Claudia Frömmel, Annemarie Brose, Jeannette Klein, Oliver Blankenstein, Stephan Lobitz

**Affiliations:** ^1^Labor Berlin-Charité Vivantes GmbH, Sylter Straße 2, 13353 Berlin, Germany; ^2^INSTAND e.V., Gesellschaft zur Förderung der Qualitätssicherung in Medizinischen Laboratorien e.V., Ubierstraße 20, 40223 Düsseldorf, Germany; ^3^Newborn Screening Laboratory, Charité-Universitätsmedizin Berlin, Augustenburger Platz 1, 13353 Berlin, Germany; ^4^Department of Pediatric Oncology/Hematology/BMT, Charité-Universitätsmedizin Berlin, Augustenburger Platz 1, 13353 Berlin, Germany

## Abstract

Sickle cell disease (SCD) does not occur in the indigenous German population, but with the increasing number of immigrants from countries at high risk for hemoglobinopathies, the question emerges whether or not a newborn screening program (NBS) for SCD disease should be initiated in Germany anyhow. We have recently shown that in Berlin, a city with a very large immigrant population, the incidence of SCD is considerable, but our findings are insufficient to make a decision for the country as a whole. In this paper we will show that a large body of epidemiological data can be generated in a relatively short period of time, with a very high degree of precision and at relatively little expense—a result that might motivate other working groups to start such a pilot project locally. We examined previously collected dried blood cards that were up to six months old, using high performance liquid chromatography (HPLC) as first method and capillary electrophoresis (CE) as second method. A single, part-time laboratory technician processed 38,220 samples in a period of 162 working days. The total costs per sample including all incidentals (as well as labor costs) were EUR 1.44.

## 1. Introduction

### 1.1. Sickle Cell Disease

SCD is one of the most common monogenetic diseases worldwide. Although it is most prevalent in Africa, in parts of the eastern Mediterranean and Asia, as a result of migration, the prevalence is also continuously increasing in central and northern Europe. SCD comprises a group of autosomal recessive *β*-hemoglobinopathies whose common feature is the occurrence of the pathognomonic hemoglobin variant hemoglobin S (HbS). Its genetic correlate is the mutation HBB:c20A>T which is found in all patients and which can occur homozygously or as compound heterozygous state in combination with a number of other mutations (e.g., HbC or *β*-thalassemia). In comparison with adult hemoglobin A (HbA) there is an amino acid substitution in the HbS molecule, with valine instead of glutamic acid in position 6 of the *β*-globin polypeptide chain. If the HbS content in an erythrocyte exceeds a critical concentration this leads under certain conditions to polymerization of deoxygenated HbS molecules, the central pathophysiological event of SCD. Through a complex process this results in acute and chronic hemolysis as well as recurrent vasoocclusive events causing pain, ischemia-reperfusion injury, and finally chronic organ damage [1].

### 1.2. Newborn Screening for Sickle Cell Disease

Numerous studies provide clear evidence that life-threatening early complications of SCD (e.g., sepsis, splenic sequestration crisis, etc.) can be largely avoided if the diagnosis is made early, that is, if possible in the first three to six months of life. Simple but very effective prophylactic measures (e.g., vaccination, penicillin prophylaxis, etc.) can then be initiated sufficiently early. This can considerably reduce morbidity and mortality [2–10]. In numerous countries SCD is therefore an established component of national NBS programs. In many other countries in which there are no national NBS programs there are at least some regional projects and pilot studies [11–30].

The physicochemical properties of HbS differ from those of the normal HbA. This phenomenon is utilized for diagnosis. All physiological hemoglobins as well as all hemoglobin variants show characteristic but not unambiguous migration patterns in all established separation methods. As in each method there are hemoglobins with the same or comparable migration characteristics it is not possible to establish conclusively which hemoglobin is present in the concrete case. This means that the results of at least two different methods have to be combined in order to be able to identify the hemoglobin present with sufficient certainty [31–34]. This rule also applies to NBS for SCD to avoid false-positive results. The methods most commonly used in NBS for SCD for separation of the hemoglobins in dried blood specimens are HPLC, isoelectric focusing (IEF), and CE [32, 35–37].

### 1.3. The German Newborn Screening Program

In Germany all newborns are entitled by law to take part in a national (so-called extended) NBS program [38]. However, participation is voluntary. The extended NBS comprises 14 congenital endocrine and metabolic disorders. It does not include SCD. In addition all newborns are screened for hearing defects. The list of disorders screened for in the German NBS program is laid down by the Federal Joint Committee (“Gemeinsamer Bundesausschuss,” G-BA). The G-BA issues the guidelines for the statutory health insurance funds (GKV) covering more than 70 million insured persons in Germany. It specifies which services in medical care are reimbursed by the GKV. If a new disease is to be included in the extended NBS program it must meet the Wilson-Jungner criteria as modified by Andermann et al. [39, 40] to fit the special conditions of the German health system. In particular there must be sufficient epidemiological data and it must have been demonstrated that early detection of the disease in question is associated with a medical and/or economic benefit.

### 1.4. Sickle Cell Disease in Germany

In Germany there are practically no epidemiological data on the incidence and prevalence of SCD. SCD has therefore not been perceived as a significant problem in the German healthcare system to date. In Berlin, a city presumed to have a high incidence, we performed a pilot project hitherto unique in Germany in which all newborns born between 1 September 2011 and 30 November 2012 who had given blood samples in the context of the extended NBS program were retrospectively additionally tested for SCD [41]. The age of the specimens presented a methodological challenge. However, two earlier publications have shown that storage for at least six weeks at room temperature or 13 months at −20°C was possible [42, 43]. In addition to generation of epidemiological data, a further goal of the project was therefore to investigate whether the dried blood specimens can be stored for longer than six weeks at room temperature and how they need to be processed after longer storage times in order to obtain reliable measurements. These are important parameters for planning similar epidemiological pilot studies.

In this paper we report on the technical aspects of our study and we discuss in more detail the special legal situation in Germany.

## 2. Methods

### 2.1. Ethics and Consent

The ethics committee of the Charité university hospital approved the study on 28 September 2011 (reference number EA2/088/11). The parents of all newborns investigated gave their written informed consent to reanalysis of blood in advance of sampling.

### 2.2. Study Design and Population

The present study is a retrospective cross-sectional investigation of an unselected cohort of newborns. All children born in Berlin between 1 September 2011 and 30 November 2012 were considered eligible for this project provided they took part in the extended NBS program. Berlin is the capital of Germany and, after London, the second most populated city in the European Union. On December 31, 2010, Berlin had a population of 3,460,725 of which 864,065 (24.97%) had a migration background. Of those, at least one half immigrated from countries with a high prevalence of hemoglobinopathies [44].

Altogether 39,249 babies were born in the study period. Of these, 95 parents or guardians did not give their consent to participation in the extended NBS. A further 422 babies were not included in the study because the parents or guardians did not consent to storage of their children's specimens beyond the time necessary for the regular screening. In 126 cases no consent was given to use of the specimens in a scientific project and in 386 cases there was too little material available to test additionally for SCD after the regular screening, so that in the end 38,220 samples could be tested.

### 2.3. Target Conditions

The primary target conditions of this study included SCD-S/S, SCD-S/C, SCD-S/D^Punjab^, SCD-S/E, SCD-S/Lepore, SCD-S/O^Arab^, SCD-S/*β*-thalassemia, SCD-S/*δ* *β*-thalassemia, and SCD-S/HPFH (hereditary persistence of fetal hemoglobin). Secondary target conditions were HbS heterozygosity, severe thalassemia, and all hemoglobin variants other than HbS in hetero- or homozygous form.

### 2.4. Blood Sampling and Logistics

The screening specimens were normally collected from the newborns in the maternity clinic or at home between the 36th and 72nd hours of life and sent to the Berlin NBS laboratory the same day in the form of dried blood spot cards (Ahlstrom 226, ID Biological Systems, Greenville, SC, USA). The Berlin NBS laboratory (Laboratory 1) is one of 11 NBS laboratories in Germany (http://www.screening-dgns.de/screening-3.php), which analyze specimens in the context of the extended NBS program, and is the only NBS laboratory in Berlin.

In newborns with a gestational age of less than 32 weeks the initial screening was followed by a second screening at a corrected age of 32 gestational weeks. Further reasons for repeat screening were performance of blood transfusions or corticosteroid or dopamine therapy before collection of the specimens for the first screening [45]. In patients screened more than once the test for SCD was performed with the last specimen obtained.

After completion of the regular screening in the context of the extended NBS program the specimens were stored at room temperature in the NBS laboratory. They were forwarded after 42 to 171 days for examination in the context of our project.

### 2.5. Measuring Methods

**Figure 1:**
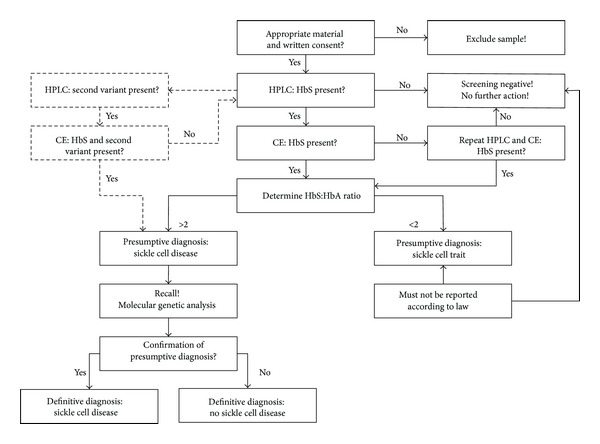
Decision tree for handling samples of the babies born in the study period (*n* = 39,249). Samples were excluded if there was no consent for screening (*n* = 95), no consent to store the sample (*n* = 422), and no consent to use the sample in a scientific project (*n* = 126), too little material (*n* = 386) or a low AUC on the HPLC machine (*n* = 4,236). Please note if any hemoglobin variant other than HbS was detected by means of HPLC, this result was also confirmed by CE.

All specimens were first examined by HPLC. Specimens with HPLC profiles consistent with SCD, sickle cell trait, *β*-thalassemia, or variant hemoglobins other than HbS were subsequently analyzed by CE as a confirmatory method [41] (Figure 1).

### 2.6. High Performance Liquid Chromatography (HPLC)

The HPLC was performed on a VARIANT nbs Newborn Screening System (Bio-Rad Laboratories, Munich, Germany) using the VARIANT nbs Sickle Cell Program. Unless expressly stated otherwise we followed the manufacturer's recommendations. The columns and all reagents such as buffers, primers, and retention time markers were purchased from the manufacturer.

A disk with a diameter of 1/8′′ (3.2 mm) was punched out of the dried blood card and placed in a well of a 96-well plate. 240 *μ*L distilled water was added per punched-out disk and the sample incubated for 21 hours (overnight) at 4–6°C. The eluate was then centrifuged for 10 minutes at 1200 g to remove the solid elements (disk, blood clots, cellulose remains, etc.) remaining in the sample. The pellet was left in the sample and the supernatant measured with evaporation protection.

The VARIANT nbs Newborn Screening System has a dual-wavelength detector, which measures the absorbance of the sample components at 415 nm. Background noise is reduced by additionally measuring each sample at 690 nm. The absorbance data are then transferred from the detector to a connected PC and converted with the software GDM 3.0 (Bio-Rad Laboratories, Munich, Germany) to a real time chromatogram in which the measured voltages (*y*-axis) are plotted against the time (*x*-axis). The software analyzes the chromatograms fully automatically using the principle of valley-to-valley integration.

In addition all chromatograms were visually inspected.

Water blanks served as negative controls and were included in every run. As controls of the preanalytical process and the automated integration and quantitation of the peaks we used eluates from dried blood spot cards from HbS and HbC carriers and retention time markers provided by the manufacturer. Interrun coefficients of variation (CV) were determined.

### 2.7. Capillary Electrophoresis (CE)

For the CE a disk 3.8 mm in diameter was punched out of the dried blood spot card and placed in a well of an 8-well strip tube. After adding 50 *μ*L distilled water the sample was incubated in the CAPILLARYS humidity chamber at 4–6°C for not less than two hours and not more than 72 hours. Then 0.6 mL of hemolysing solution was added. Measurement of the hemolysate was then performed on a CAPILLARYS 2 CE system. In each run controls with hemoglobins A, F, S, and C provided by the manufacturer were measured. Data acquisition, management, and analysis were conducted with the PHORESIS 6.51 software. All material for the CE was purchased from Sebia, Fulda, Germany.

### 2.8. Handling of Results

If both HPLC and CE analyses of a baby's blood were indicative of SCD, the family was contacted and referred to our department of pediatric hematology, where the presumed diagnosis was confirmed from a fresh blood sample by molecular genetic analysis. Telephone numbers and addresses were extracted from the routine NBS database. Carrier states were not reported as required by our local ethics committee.

## 3. Results

**Figure 2:**
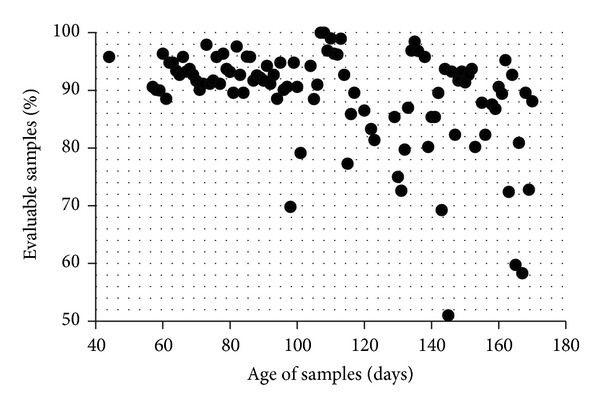
Each dot represents the mean of an average of 353 samples (range 24–1090) of the same age. Of the samples that were analyzed within three months after being taken normally more than 90% were evaluable.

A maximum of four 96-well plates was measured per day and 455 plates were measured in 180 runs. Six column lots were used during the entire study. Altogether 38,220 samples were screened by HPLC, 34,084 of which were subject to evaluation. 4,236 samples had to be excluded because the corresponding chromatograms showed a low total AUC. As expected, the percentage of samples with a low AUC was positively correlated with sample age (Figure 2). Older samples showed more degradation of hemoglobin resulting in more background noise and less analyzable material. Older samples also contained more filter paper lint, which interfered with the analysis by causing technical problems such as pressure increases or detector errors. In one case we observed a retention time shift. With 74 of our oldest 84 samples (88.1%) we obtained chromatograms with an AUC > 900,000 *μ*Vs, although they had been taken from dried blood spot cards 171 days previously. The samples thus fulfilled the minimum criteria for testing which we defined as “adequate quality.” Nevertheless the proportion of unusable results increased significantly when the dried blood cards were more than three months old (Figure 2).

### 3.1. Screening-Positive Newborns

**Figure 3:**
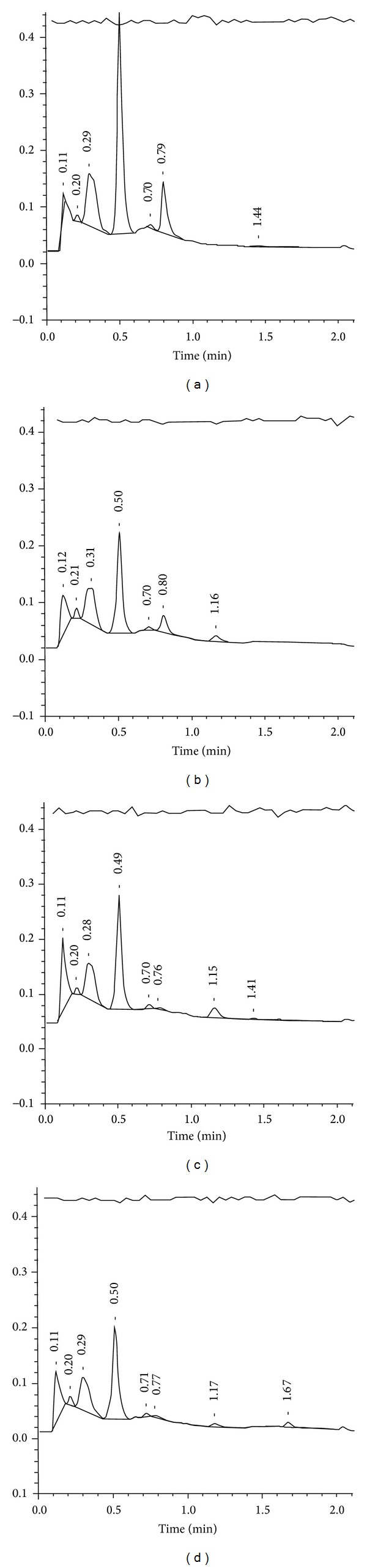
Representative HPL chromatograms with the presumed hemoglobin patterns FA (a), FAS (b), FS (c), and FSC (d).

Of 34,084 samples included, 14 samples showed an HPLC pattern consistent with the diagnosis of SCD. 10 of which were interpreted as FS; that is, only hemoglobins F and S were assumed to be present. An FS pattern indicates homozygosity for HbS as well as compound heterozygosity for HbS and *β*-thalassemia or *δ* *β*-thalassemia or HPFH, respectively. In four newborns we detected an FSC pattern; that is, only hemoglobins F, S, and C were present in these babies. This is only explainable by compound heterozygosity for HbS and HbC clinically resulting in SCD-S/C (Figure 3).

CE was used to corroborate HPLC results. In all 14 cases, CE patterns were consistent with the corresponding HPLC results. Consequently, these newborns were considered “screening positive” and appointed to our pediatric hematology outpatient clinic to finally confirm diagnoses (Table 1). We did not observe false-positive findings.

### 3.2. Screening-Negative Newborns

The remaining 34,070 newborns showed hemoglobin patterns without any evidence of any phenotype of SCD and were thus classified as “screening negative.” Among these we identified a total of 236 newborns who were heterozygous for a hemoglobin variant. This was HbS in 165 cases, HbE in 27, HbC in 23, and HbD in 11. In ten further cases there was a variant other than HbS, E, C, or D. In the cases interpreted as heterozygosity for HbS the HbS : HbA ratio was <2.0.

Until to date, it has not come to our attention that in any of these screening-negative babies the diagnosis of SCD has been made afterwards. However, these children are now between 14 and 28 months of age and follow-up is not systematically organized. So it is possible that screening false-negative subjects, for example, children suffering from milder subtypes of SCD like SCD-S/*β* ^+^ thalassemia, are missed.

### 3.3. Precision of HPLC

Retention time markers (RTM) were provided by the manufacturer. RTM are lyophilized controls containing hemoglobins F, A, E, and S and hemoglobins F, A, D, and C, respectively, to monitor the precise separation of the aforementioned hemoglobins by the HPLC instrument [46]. RTM help to detect instabilities in pump pressure or gradient composition which is an indispensable requirement for correct analytical performance, because raw data are processed electronically and the correct interpretation and integration of peaks appearing in particular time windows require the full functionality of the hardware.

If the RTM indicated a functional disorder of the HPLC machine, we repeated the runs that were probably affected.  Table 2 shows our results of several measurements (*n* = 130) of the retention time markers in comparison to the manufacturer's specifications. Our data show a high precision of retention times (CV ≤ 1.5%). The maximum deviation from mean retention time was ±0.042 min for HbS.

Table 3 shows the mean and the range of retention times of the various hemoglobins detected in the dried blood spot samples of our study population. The retrospective analysis of these retention times provides information about the stability of the HPLC system over the whole study period with this challenging material. The CV was ≤ 1% for all main hemoglobins (A, S, E, D, and C).

As many factors during the whole analytical procedure may affect test results, dried blood samples from five newborns, found to be heterozygous for either HbS or HbC, respectively, were established as additional quality controls. These samples were randomly integrated into several runs including all steps from pre- to postanalytics to include all influencing factors.  Table 4 shows the results of repeated analyzes of these quality control samples known to be heterozygous for HbS (A) or HbC (B) on different working days.

### 3.4. Precision of CE

As quality controls of CE liquid control samples containing hemoglobins F, A, S, and C were used in each run on different working days (Table 5).

### 3.5. Costs

All in all we examined samples from 38,220 newborns, 37,970 only by HPLC, 250 additionally by CE, because the samples showed an abnormal hemoglobin pattern in the HPLC. For the examination of the entire cohort we incurred costs of EUR 55,000. A large part of this sum (just under 50%) was labor costs. However, the sum also contains the costs for instrument hire, for consumables, and so forth. This translates into a per-sample cost of EUR 1.44 (US$1.97).

## 4. Discussion

There are practically no epidemiological data to date on the prevalence and incidence of SCD in Germany [41, 47, 48]. Cautious estimates made so far suggest a figure of about 3000 to 4000 patients in the whole country. These patients are without exception of immigrant origin [47].

Over a period of more than one year we tested, without preselection on the basis of ethnic origin, 87% of all children born in Berlin (34,084 of 39,249) for the presence of SCD and identified 14 with the disease [41]. The incidence of SCD (4.11/10,000) amongst the children examined was thus higher than the incidence of any other endocrine or metabolic disorder tested for in the context of the German extended NBS program. SCD should therefore be included in the NBS at least in Berlin. In order to decide whether there is a need for a national NBS program for SCD we now require data from other regions, particularly from presumed low-prevalence areas.

The results presented in this paper show that such epidemiological data can be generated with investment of relatively little time and money as the samples do not need to be examined fresh on a day-to-day basis. Instead, large numbers of samples can be analyzed retrospectively. It is clear that the costs per sample (1.44 EUR) in the context of our pilot study will not be comparable with the expenses in the context of a national screening program. While the costs for consumables, and so forth, would probably be a little lower on account of the substantially higher patient numbers, there would be considerable additional expenses for administrative purposes such as quality assurance. Further additional costs would arise for staff training and for the entire infrastructure, which has to be made available around the actual testing.

In this study dried blood spot cards up to six months old provided satisfactory results. However, very old samples present a particular challenge because cellulose particles, which come off the dried blood cards together with the blood, can interfere considerably with the measurement. They are deposited in the fine capillaries of the measuring instruments, clogging them or creating additional, unspecific measuring signals. The continuously increasing degradation of the hemoglobin also leads to additional measuring signals, particularly to increased background noise, as well as reducing the amount of analyzable material. In the worst case, one might even miss a significant disease due to misinterpretation of a peak resembling HbA; for example, a *β*-thalassemia major pattern could be confused with a physiological FA pattern or homozygosity for HbS could be confused with SCT. Sample preparation is therefore of decisive importance.

Factors, which can influence the sample quality, are as follows.

1. The concentration of the hemoglobin in the hemolysate. This depends on the volume of the blood sample, thus on the saturation of the filter card and on the degree of degradation of the hemoglobin. The latter is essentially dependent on the age and the storage conditions of the sample. Further important parameters are the elution volume and elution time.
2. Instrument performance is influenced significantly by the condition of the dried blood spot card. This is in turn also dependent on the sample age and the storage conditions. In the case of older cards cellulose particles are removed from the card together with the blood and can then interfere with the measurement.

Prolonging the elution time increases the hemoglobin concentration in the hemolysate and also the likelihood of cellulose particles becoming detached from the filter card. The elution conditions described in the methods section take into account both phenomena and represent the necessary compromise.

The implementation of pilot studies of this kind requires the availability of a high throughput method. In our experience, HPLC and CE are equally suitable. The CE electropherograms appeared to us to be easier to interpret than the HPLC chromatograms because there were fewer unspecific signals in the former. Whichever method is chosen, it is crucial that the analytical laboratory has special expertise with regard to the IT-supported interpretation of the raw measurements.

Disease screening programs are characterized by the fact that certain people are examined for the presence of a certain disease simply because they belong to a certain population. The subjects are examined or tested regardless of whether there is any concrete reason to suspect the presence of the target disease. The large majority of the subjects screened are completely healthy. This raises particular ethical, legal, technical, and economic issues.

The German Genetic Testing Act (GenDG) came into force on January 2, 2010 and is a constant topic of debate on account of its very strict regulations [49]. It controls genetic testing in humans and the use of genetic samples and data. The GenDG specifies, interalia, that analyses of the products of nucleic acids (gene product analyses) are also considered genetic analyses and therefore deemed legally equal to examination of the number and structure of the chromosomes and of the molecular structure of DNA and RNA. This means that hemoglobinopathy testing using protein separation methods such as HPLC or CE comes under the GenDG.

The ultimate goal of the GenDG is to prevent the potential dangers and discrimination associated with the examination of human genetic traits. One of the most important principles is the right of the individual to informational self-determination. This right, which enjoys internationally unique protection in Germany, includes the right to know and, more important in this context, the right not to know. Its most important implication is that we are not allowed in Germany to report sickle cell trait.

The GenDG distinguishes between genetic tests performed in a medical context and tests performed in a research context and only applies to tests ordered in a medical context. A distinction is also made between diagnostic tests and predictive tests. In addition, the GenDG regulates genetic screening, which would include NBS for SCD. German law requires the establishment of an independent, interdisciplinary Genetic Testing Commission (GEKO), which has been set up at the Robert Koch Institute and, amongst other things, lays down the requirements for the performance of genetic screening tests [49]. These requirements are based on the specific criteria for genetic screening developed by Andermann et al. on the basis of the WHO screening criteria of Wilson and Jungner [39, 50]. The GEKO has expressly stated that results of studies performed in other countries with different healthcare systems and different population structures cannot necessarily be extrapolated to Germany [51]. For example, some screening criteria are based on local conditions, which do not obtain in Germany or at least not in all parts of the country. Sickle cell patients cannot, in particular, be offered state-of-the-art care everywhere in Germany. Nonmalignant hematology has been almost entirely neglected in the training of all groups of healthcare professionals in the past and the hemoglobinopathies as a whole are still not seen as a problem in Germany. However, a national strategy for prevention and treatment of SCD is currently under development. The Society for Pediatric Oncology and Hematology (GPOH) has commissioned the establishment of a patient register, reference centers, and a treatment guideline. This is important because, if it is to make any sense, a newborn screening program requires not only the identification of sick babies but also a functioning infrastructure, which ensures that affected children also receive appropriate care after the diagnosis. This includes both medical and psychosocial care as well as sufficient counseling resources for the patients and their parents.

## 5. Conclusions

Our pilot project shows that it makes sense, at least regionally, to think about the introduction of some kind of NBS program for SCD in Germany, whatever specific form this takes. The pilot projects necessary for this must be embarked on now. In addition to the presumed high-prevalence regions such as Berlin, Hamburg, the Ruhr region, or Frankfurt, epidemiological data should also be collected from presumed low-prevalence regions (e.g., rural Bavaria and Baden-Württemberg).

Our data show that the organization and realization of such pilot projects is relatively unproblematic and inexpensive as samples can be collected over several months and then analyzed together. However, we recommend that the samples should not be older than three months at the time of analysis. It is of fundamental importance that the Genetic Testing Act does not apply to such pilot projects as they involve genetic testing in the context of research.

## Figures and Tables

**Table 1:** Detailed information on screening-positive babies.

Patient number (ethnical origin)	Screening patterns HPLC/CE	Molecular genetic result
# 1 (West African)	FSa/FS	SCD-S/S
# 2 (West African)	F5SaB/FS	SCD-S/S
# 3 (Middle East)	FSa5B/FS	SCD-S/*β* ^0^ thalassemia
# 4 (West African)	FaCS/FSC	SCD-S/C
# 5 (Middle East)	F4SA/FS	SCD-S/S
# 6 (West African)	FSCa/FSC	SCD-S/C
# 7 (Middle East)	FSa5/FS	SCD-S/S
# 8 (West African)	FSaB/FS	SCD-S/S
# 9 (West African)	FS4a/FS	SCD-S/S
# 10 (West African)	FS5aB/FS	SCD-S/S
# 11 (West African)	FCSa/FSC	SCD-S/C
# 12 (West African)	FSa2B/FS	SCD-S/S
# 13 (West African)	FSa2B/FS	SCD-S/S
# 14 (West African)	FCS1/FSC	SCD-S/C

**Table 2:** High interrun precision of HPLC retention times of retention time markers.

Hemoglobin	Retention times of RTM mean (range) [min]	CV [%]	Manufacturer's specifications mean (range) [min]
F	0.494 (0.471–0.510)	1.5	0.530 (0.470–0.590)
A	0.792 (0.770–0.804)	0.9	0.800 (0.730–0.870)
E/A2	0.954 (0.919–0.970)	1.0	0.970 (0.930–1.010)
D	1.042 (1.005–1.072)	1.1	1.060 (1.010–1.110)
S	1.158 (1.116–1.183)	1.2	1.190 (1.130–1.250)
C	1.663 (1.634–1.684)	0.6	1.680 (1.610–1.750)

**Table 3:** High precision of HPLC retention times of hemoglobins derived from dried blood spot samples.

Presumptive hemoglobin	Retention times mean (range) [min]	CV [%]	n
A	0.800 (0.785–0.816)	0.8	100
E	0.952 (0.936–0.968)	0.8	26
D	1.044 (1.028–1.060)	1.0	11
S	1.154 (1.190–1.130)	0.9	100
C	1.660 (1.647–1.670)	0.4	23

**(a):** 

	HbF mean [%]	CV [%]	HbA mean [%]	CV [%]	HbS mean [%]	CV [%]	*n*
Control 1	37.3	2.9	8.9	5.3	3.6	6.3	11
Control 2	40.1	4.8	1.9	27	1.0	50	8

**(b):** 

	HbF mean [%]	CV [%]	HbA mean [%]	CV [%]	HbC mean [%]	CV [%]	*n*
Control 3	39.6	5.7	1.7	22	1.3	28	11

Precision was reduced if certain hemoglobin fractions were present in an order close to the detection limit of 1% of the total AUC.

**Table 5:** Interrun precision of CE determined on liquid control samples provided by the manufacturer.

	HbF mean [%]	CV [%]	HbA mean [%]	CV [%]	HbS mean [%]	CV [%]	HbC mean [%]	CV [%]	*n*
Control FASC	18.8	4.8	35.5	2.0	14.5	4.3	5.4	7.7	5
